# Analysis of the influence of a solenoid magnetic field in the azimuth transmission system

**DOI:** 10.1038/s41598-021-95783-0

**Published:** 2021-08-10

**Authors:** Zhiyong Yang, Junchen Song, Wei Cai, Gaoxiang Lu, Zhiwei Zhang

**Affiliations:** Xi’an Research Institute of High-Tech, Xi’an, 710000 Shaanxi China

**Keywords:** Magneto-optics, Engineering

## Abstract

A solenoid magnetic field plays an important role in a non-line-of-sight azimuth transmission system based on polarization-maintaining fiber, which is directly related to the transmission accuracy of azimuth information. This research mainly studies the factors that affect the solenoid magnetic field according to the modulation signal from the direct current to the alternating current, as well as the hollow solenoid. First, the magnetic field components of the static solenoid are derived from the Biot–Savart law by using the uniform cylindrical current equivalent model. Then, the magnetic field of the near axial region is studied from the axial and radial directions, and the feasibility of calculating the magnetic field of the multi-layer solenoid with the superposition principle is verified by measuring the magnetic field of each position on the axis of the solenoid with a Gauss meter. Finally, the alternating electromagnetic field model is established using Maxwell’s equations, and the magnetic and electric fields of the hollow solenoid are further solved. The results show that the magnetic field in the middle part of the magneto-optic glass is more stable, and the magnetic collecting ability of the solenoid is stronger. The magnetic field intensity at the center of the magneto-optic modulation solenoid of the system is the largest, and it decreases with the distance from the center. The alternating electromagnetic field is closely related to frequency. The results provide a reference for the study of the azimuth accuracy of a non-line-of-sight azimuth transmission system.

## Introduction

A non-line-of-sight azimuth transmission system based on polarization-maintaining fiber (PMF), combined with the Marius law for detecting an azimuth signal, can theoretically measure the angle between two or more devices without a mechanical connection for the condition of intervisibility, and this type of system is widely used in many fields, such as the initial alignment of a spacecraft launch, instrument measurement and tunnel engineering^1^.

Magneto-optical modulation technology based on the Faraday effect has attracted increasing attention due to its strong anti-interference ability and high precision. The essence of magneto-optical modulation technology is the use of the Faraday effect to modulate and demodulate a linearly polarized light signal carrying azimuth information to improve the accuracy of azimuth transmission^2^. In the process of magneto-optic modulation, an alternating current signal is used to drive the solenoid magnetic field, which generates the polarization plane polarized light deflection, Faraday rotation, and implementation of the polarized light signal modulation. Therefore, the accuracy of information on the alternating current that is used to drive the solenoid magnetic field to the other side of the transmission is critical. The current analyses of the solenoid magnetic field for most literature references are focused on practical applications^3–6^. For example, the National University of Defense Technology has built a solenoid magneto controlled thermal protection system for the thermal protection of hypersonic aircraft^7^. Kulikov's team discussed the design of a superconducting shield and carried out experimental and numerical studies on the magnetic field uniformity of a solenoid with superconductivity^8^. Some studies have focused on the distribution of a solenoid magnetic field^9–11^, and some studies have briefly analyzed a solenoid magnetic field^12,13^. However, few studies have focused on the paraxial region of the solenoid and the solenoid alternating magnetic field in a non-line-of-sight azimuth transmission system.

In this research, a non-line-of-sight azimuth transmission system based on polarization-maintaining fiber is taken as the object. First, the principle of the system and the factors restricting the accuracy of the azimuth transmission are analyzed and explained. Then, the components of the solenoid magnetic field in the axial, circumferential and radial directions are deduced theoretically. Furthermore, the magnetic field distribution in the paraxial region and the magnetic field characteristics of multi-layer solenoids are analyzed, and the electromagnetic field model of a hollow solenoid driven by alternating current is established based on an equation.

## System principle

**Figure 1:**
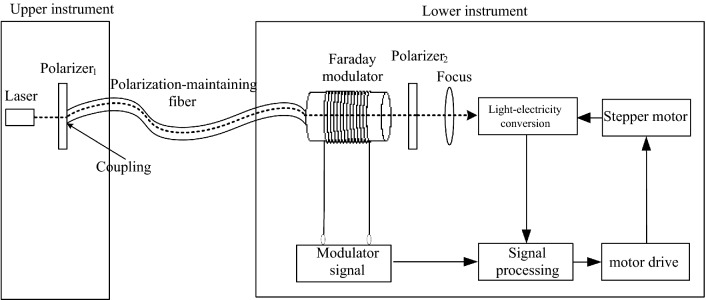
Schematic diagram of azimuth transmission system based on polarization-maintaining fiber.

Figure 1 show the schematic diagram of the non-line-of-sight azimuth transmission system based on polarization-maintaining fiber. As shown in Fig. 1, the upper and lower instruments are installed in non-line-of-sight positions. The two instruments are connected by a polarization-maintaining fiber. The laser emitted from the laser in the upper instrument passes through the polarizer and becomes linearly polarized light, and the linearly polarized light carrying the polarizer azimuth information is coupled into the polarization-maintaining fiber and transmitted to the modulator of the lower instrument. When the linearly polarized light passes through the magneto-optic material in the modulator, the polarized light signal is modulated by the alternating magnetic field driven by the modulated signal. The modulated polarized light signal is processed with a polarizer, a focusing mirror, photoelectric conversion, signal detection, and processing. The electric signal corresponding to the azimuth angle is extracted, and the instrument is driven to rotate to complete the azimuth synchronization. The azimuth information transmission is achieved under a non-intervisibility condition^1^.

Using the Jones matrix transformation with Maxwell's method, the light intensity signal emitted through the magneto-optic modulator is as follows:

1 $$I = \frac{1}{4}I_{0} \left[ {1 - \cos \left( {m_{f} \sin \omega t} \right)\cos 2\alpha - \sin \left( {m_{f} \sin \omega t} \right)\sin 2\alpha \cos \delta } \right],$$

where $I_{0}$ is the light intensity after passing through the polarizer,$m_{f}$ is the magneto-optical modulation,$\alpha$ is the azimuth angle,$\omega$ is the frequency of the sinusoidal excitation signal on the modulation coil, and $\delta$ is the phase difference caused by the fiber polarization mode birefringence.

With $\cos (m_{f} \sin \omega t)$ and $\sin (m_{f} \sin \omega t)$, the Bessel functions of the first kind are extended and terms of order higher than the second order are ignored. The electrical signal converted by photoelectricity is

2 $$u \approx ku_{0} \left[ {1 - J_{0} \left( {m_{f} } \right) \cdot \cos 2\alpha - 2J_{1} \left( {m_{f} } \right) \cdot \sin 2\alpha \cos \delta \sin \omega t - 2J_{2} \left( {m_{f} } \right) \cdot \cos 2\alpha \cos (2\omega t)} \right],$$

where $u_{0} = \eta I_{0} /4$, $\eta$ is the photoelectric conversion coefficient, $k$, is the magnification factor, and $J$ is the Bessel function of the first kind.

After setting $U = 2 \cdot ku_{0} \cdot J_{1} \left( {m_{f} } \right) \cdot \sin \left( {2\alpha } \right)$ and $V = 2 \cdot ku_{0} \cdot J_{2} \left( {m_{f} } \right) \cdot \cos \left( {2\alpha } \right)$, the mixed signal $u$ is filtered by a direct filter, and the alternating current (AC) signal is obtained:

3 $$u_{A} = - U\cos \delta \sin \omega t - V\cos (2\omega t).$$

It is found that there are always two extreme points in the AC signal $u_{A}$, the abscissa of which do not change with the change of the azimuth angle, nor do they change with the change of the phase difference. The sampling integration circuit is used to collect the extreme points $u_{A1}$ and $u_{A2}$:

4 $$\left\{ {\begin{array}{*{20}c} {u_{A1} = 2ku_{0} \left[ {J_{2} \left( {m_{f} } \right)\cos 2\alpha - J_{1} \left( {m_{f} } \right)\cos \delta \sin 2\alpha } \right]} \\ {u_{A2} = 2ku_{0} \left[ {J_{2} \left( {m_{f} } \right)\cos 2\alpha + J_{1} \left( {m_{f} } \right)\cos \delta \sin 2\alpha } \right]} \\ \end{array} } \right..$$

The extreme points $u_{A1}$ and $u_{A2}$ are processed by ‘subtraction division and summation’ to obtain the azimuth angle solution model:

5 $$\alpha = \frac{1}{2}\arctan \left[ {\sec \delta \frac{{J_{2} \left( {m_{f} } \right)}}{{J_{1} \left( {m_{f} } \right)}} \cdot \frac{{u_{A2} - u_{A1} }}{{u_{A2} + u_{A1} }}} \right].$$

It can be seen from Eq. (5) that both the signals $u_{A1}$ and $u_{A2}$ acquired by the sampling integral and the solution model itself contain the first-order Bessel function $J_{1} \left( {m_{f} } \right)$ and the second-order Bessel function $J_{2} \left( {m_{f} } \right)$ with an $m_{f}$ as the parameter. In addition, $L$ is a fixed quantity that can be directly measured. The Verdet constant $V$ and the magnetic induction intensity $B$ of the material are very important, and they are affected by many factors, including the material itself, the solenoid structure, the modulation parameters, and the inevitable temperature rise. They directly affect the output signal, and ultimately affect the measurement of misalignment angle. Moreover, this may cause signal distortion and result in significant deviation in the angle measurement result.

The solenoid is electrified to produce a magnetic field, which determines the Faraday rotation angle. According to the Biot–Savart law, the magnetic field can be calculated theoretically when the coil density $n$ and the driving current $I$ are known, but these two parameters are not the only factors that determine and affect the magnetic field. The temperature rise is also one of the factors that increase the impedance, and when there is a medium, the magnetic induction $B$ inside the solenoid varies from medium to medium, and this difference is affected by the size of the external magnetic field, the frequency, and the temperature. Therefore, if the medium near the measurement point is selected for the direct measurement, the results will be slightly different from the real magnetic field intensity. Therefore, the solenoid magnetic field is still a complex problem that cannot be treated simply according to the traditional method. The magnetic induction $B$ is a main parameter that restricts the accuracy.

## Analysis of static magnetic field of solenoid

According to the analysis of the factors affecting the accuracy of the magneto-optical modulation described in the system principle, it is known that the solution of the solenoid static magnetic field lies in studying the magnetic field in the solenoid. This section mainly describes the study of the size, direction, and distribution of the magnetic field components. Based on the research for a solenoid static magnetic field, the solenoid magnetic field excited by alternating current can be further studied.

### Theoretical derivation of magnetic field

**Figure 2:**
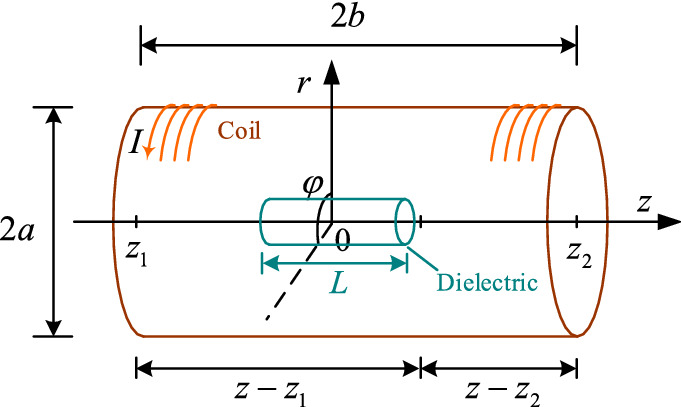
Schematic diagram of the solenoid coil.

A single-layer solenoid with a thin wire tightly wound is set. Its radius is $a$, its length is $2b$, the number of turns are $N$, the current $I$ is applied, and the coil density is defined as $n = N/2b$. The solenoid center is taken as the origin, and the central axis is taken as the $z$ axis to establish the cylindrical coordinate system $\left( {r,\varphi ,z} \right)$, as shown in Fig. 2. The positive direction of the $z$ axis and the current flow direction of the coil satisfy the right-handed spiral relationship. Considering the close winding, the line current is equivalent to the surface current along the cylindrical surface.

Any point ${\text{Q}}\left( {a,\varphi ^{\prime},z^{\prime}} \right)$ on the current distribution plane in Fig. 2 and any field point ${\text{P}}\left( {r,\varphi ,z} \right)$ in the cylindrical coordinate space are taken. Then, the area current density vector at the source point ${\text{Q}}$ is:

6 $${\mathbf{J}}_{s} \left( {\text{Q}} \right) = nI\left( { - \sin \varphi ^{\prime}{\mathbf{i}} + \cos \varphi ^{\prime}{\mathbf{j}}} \right).$$

The vector path from source point ${\text{Q}}$ to field point ${\text{P}}$ is

7 $$\begin{aligned} {\mathbf{R}}\left( {\text{Q,P}} \right) = & \left( {x - x^{\prime}} \right){\mathbf{i}} + \left( {y - y^{\prime}} \right){\mathbf{j}} + \left( {z - z^{\prime}} \right){\mathbf{k}} \\ = & \left( {r{\cos}j - a{\cos}j^{\prime}} \right){\mathbf{i}} + \left( {r{\sin}j - a{\sin}j^{\prime}} \right){\mathbf{j}} + \left( {z - z^{\prime}} \right){\mathbf{k}}. \\ \end{aligned}$$

The module of the vector path is

8 $$D = |{\mathbf{R}}\left( {\text{Q,P}} \right)| = \left[ {r^{2} + a^{2} - 2ar\cos \left( {\varphi - \varphi^{\prime}} \right) + \left( {z - z^{\prime}} \right)^{2} } \right]^{1/2} .$$

The expression of the vector product of the current density vector and the ${\text{QP}}$ vector path diameter in cylindrical coordinates is

9 $${\varvec{J}}_{{\varvec{s}}} \left( {\text{Q}} \right) \times {\mathbf{R}}\left( {\text{Q,P}} \right) = nI\left[ {\left( {z - z^{\prime}} \right)\cos \theta \cdot {\mathbf{r}} - \left( {z - z^{\prime}} \right)\sin \theta \cdot {\mathbf{\varphi }} + \left( {a - r\cos \theta } \right) \cdot {\mathbf{z}}} \right].$$

where ${\mathbf{r}}$, ${\mathbf{\varphi }}$, and ${\mathbf{z}}$ are the unit vectors along the radial, circumferential, and axial directions, respectively, in the cylindrical coordinate system, and $\theta = \varphi - \varphi ^{\prime}$. According to the Biot–Savart law, the magnetic induction intensity of the solenoid coil at point ${\text{P}}$ can be calculated as

10 $$\begin{gathered} B\left( {r,\varphi ,z} \right) = \frac{{\mu_{0} }}{4\pi }\int_{s} {\frac{{{\varvec{J}}_{{\varvec{s}}} \left( Q \right) \times {\varvec{R}}\left( {Q,P} \right)}}{{D^{3} }}dS\left( Q \right)} \\ = \frac{{\mu_{0} naI}}{4\pi }\left[ {\int_{ - \pi }^{\pi } {d\theta \int_{{z_{1} }}^{{z_{2} }} {\frac{{\left( {z - z^{\prime}} \right)\cos \theta }}{{D^{3} }}dz^{\prime}{\mathbf{r}} + \int_{ - \pi }^{\pi } {d\theta \int_{{z_{1} }}^{{z_{2} }} {\frac{{ - \left( {z - z^{\prime}} \right)\sin \theta }}{{D^{3} }}dz^{\prime}{\mathbf{\varphi }} + \int_{ - \pi }^{\pi } {d\theta \int_{{z_{1} }}^{{z_{2} }} {\frac{a - r\cos \theta }{{D^{3} }}dz^{\prime}{\mathbf{z}}} } } } } } } \right]. \\ \end{gathered}$$

where $D$ is the module of the vector path, $\mu_{0}$ is the vacuum permeability. Based on Eq. (10), the components of the solenoid magnetic field in the three directions can be obtained.

1. Axial component

The axial component of the solenoid magnetic field is

11 $$B_{z} \left( {r,\varphi ,z} \right) = \frac{{\mu_{0} nIa}}{4\pi }\int_{ - \pi }^{\pi } {\text{d}} \theta \int_{{z_{1} }}^{{z_{2} }} {\frac{a - \cos \theta }{{D^{3} }}{\text{d}}z^{\prime}} = \frac{{\mu_{0} nI}}{2\pi }\left[ {f_{z} \left( {r,z - z_{2} } \right) - f_{z} \left( {r,z - z_{1} } \right)} \right],$$

where, $f_{z} \left( {r,t} \right) = \frac{t}{{\sqrt {\left( {r + a} \right)^{2} + t^{2} } }}\left[ {\frac{r - a}{{r + a}}\prod \left( {h,g} \right) - {\text{K}}\left( g \right)} \right]$ and $g = \sqrt {\frac{4ar}{{\left( {r + a} \right)^{2} + t^{2} }}}$, and $h = - \frac{4ar}{{\left( {a + r} \right)^{2} }}$.${\text{K}}\left( g \right) = \int_{0}^{{\frac{\pi }{2}}} {\frac{d\alpha }{{\sqrt {1 - g^{2} \sin^{2} \alpha } }}}$ and $\prod \left( {h,g} \right) = \int {\frac{d\alpha }{{\left( {1 + h\sin^{2} \alpha } \right)\sqrt {1 - g^{2} \sin \alpha } }}}$ are the first and the third kinds of total elliptic integrals, respectively.

2. Radial component

The radial component of the solenoid magnetic field is

12 $$B_{r} \left( {r,\varphi ,z} \right) = \frac{{\mu_{0} nI}}{2\pi }\sqrt{\frac{a}{r}} \left[ {f_{r} \left( {r,z - z_{2} } \right) - f_{r} \left( {r,z - z_{1} } \right)} \right] = \frac{{\mu_{0} nI}}{2\pi }\sqrt{\frac{a}{r}} \left[ {f\left( {g_{2} } \right) - f\left( {g_{1} } \right)} \right],$$

where $f\left( g \right) = \left( {\frac{2}{g} - g} \right){\text{K}}\left( g \right) - \frac{2}{g}{\text{E}}\left( g \right)$, and $g_{1} = \sqrt {\frac{4ar}{{\left( {r + a} \right)^{2} + \left( {z - z_{1} } \right)^{2} }}}$, and $g_{2} = \sqrt {\frac{4ar}{{\left( {r + a} \right)^{2} + \left( {z - z_{2} } \right)^{2} }}}$.

3. Circumferential component

The circumferential component of the solenoid magnetic field is

13 $$B_{\varphi } \left( {r,\varphi ,z} \right) = - \frac{{\mu_{0} nI}}{4\pi }\int_{{z_{1} }}^{{z_{2} }} {\left( {z - z^{\prime}} \right)\left( {\int_{ - \pi }^{\pi } {\frac{\sin \theta }{{D^{3} }}{\text{d}}\theta } } \right)} {\text{ d}}z^{\prime} = 0,$$

It can be seen from Eqs. (11) and (12) that the expressions of the axial component $B_{z}$ and the radial component $B_{r}$ are independent functions of $\varphi$. so, they can also be written as $B_{z} \left( {r,z} \right)$ and $B_{r} \left( {r,z} \right)$, which fully reflects the cylindrical symmetry of the solenoid. As can be seen from Eq. (13), there is no circumferential component in the solenoid magnetic field because the model uses a uniform cylindrical surface current equivalent, which is equivalent to the superposition of multiple ring currents in the axial direction. The current has a component along the $\varphi$ circumferential direction but not in the $z$ axial direction. The circumferential magnetic field produced by the axial component of the current should be considered when the pitch of the solenoid is obvious, but the application of this kind of solenoid is rare.

### Distribution characteristics of magnetic field in the paraxial region

In addition to calculating the magnitude of the point magnetic field, it is sometimes necessary to consider the distribution of the overall magnetic field in a certain space. For example, in the magneto-optical modulation system, when the magneto-optical material is installed, it is required to be coaxial with the solenoid at first and then at the center of the solenoid. At this time, the light emitted by the laser propagates along the central axis, passes through the magneto-optical glass, and forms a slender optical column with the length of glass. The range of the optical column is the effective area of the interaction between the magnetic field and the light, which is called the paraxial region. When studying the magnetic field in the paraxial region, its size and distribution are analyzed based on the axial and radial components.

1. Axial magnetic field

Setting $r = {0}$, based on the symmetry of the solenoid, it can be seen that the radial component of the magnetic field on the axis is zero, that is, $B_{r} \left( {0,z} \right) = 0$, and only the axial component is considered. In order to facilitate the analysis, the geometric center of the solenoid is taken as the coordinate origin, that is, $z_{2} = - z_{1} = b$, and the axial component of the magnetic field on the central axis is obtained by simplifying Eq. (11) as follows:

14 $$B_{z} \left( {0,z} \right) = \frac{{\mu_{0} nI}}{2}\left( {\frac{z + b}{{\sqrt {a^{2} + \left( {z + b} \right)^{2} } }} - \frac{z - b}{{\sqrt {a^{2} + \left( {z - b} \right)^{2} } }}} \right),$$

Equation (14) is the expression of the magnetic field on the axis of the solenoid, which is distributed on the $z$-axis, has only $z$ components, and is related to the radius $a$ and half-length $b$ of the solenoid. When $b \gg a$ is satisfied, Eq. (14) is further reduced to

15 $$B_{z} \left( {0,z} \right) \approx \mu_{0} nI = B_{\infty } .$$

Equation (15) is the expression of the magnetic field of the infinite solenoid. Here, the magnetic field on the central axis is only related to the coil density and the current, and the magnetic field is $\mu_{0} nI$. It should be noted that infinite length does not exist, but for a long straight solenoid with $b > {10}a$, it is very convenient to analyze the magnetic field with infinite length.

**Figure 3:**
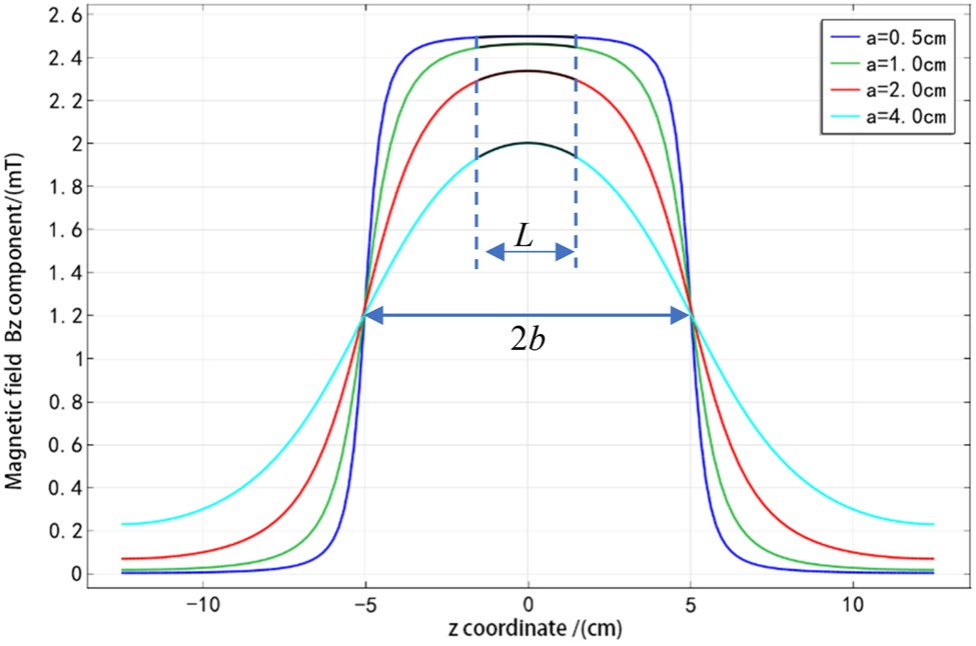
Distribution of $B_{z}$ component on the axis when $b$ is constant and $a$ is changing.

For the convenience of discussion, the aspect ratio $m$ is defined as $m = b/a$. The current $I = {2 }{A}$, coil density $n = {1000}$, solenoid length ${2}b = {\text{10 cm}}$ and radius $a = {0}{\text{.5 cm}}$, ${1}{\text{.0 cm}}$, ${2}{\text{.0 cm}}$, and ${4}{\text{.0 cm}}$ are taken, and $r = {0}$ is set. The size and distribution of $B_{z}$ are shown in Fig. 3.

It can be seen from Fig. 3 that when the solenoid length $b$ is constant, the axial magnetic field $B_{z}$ inside the solenoid increases with the decrease of the radius $a$, while the magnetic field outside the solenoid decreases. The smaller the radius $a$, the more stable the magnetic field in the middle section $\left( { - L/2 < z < L/2} \right)$ of the magneto-optical glass. However, the attenuation of the magnetic field outside the tube is faster. This shows that the larger the ratio of length to diameter $m$, the stronger the magnetic concentrating ability of the solenoid, and the better the uniformity of the internal magnetic field. The uniformity here can be explained by the fact that the size of the magnetic field is almost constant in the range $- L/2 < z < L/2$, indicating that the uniformity is better. $L$ is the length of the magneto-optical medium.

2. Radial magnetic field

The radius of the solenoid $a = {\text{1 cm}}$, the half-length $b = {5}\,{\text{ cm }}$, the coil density $n = {1000}$, the current $I = {\text{2 A}}$, and the components $z_{1}$ and $z_{2}$ of Eq. (12) with $- b$ and $b$ are taken separately. The different $r(r < a)$ values are taken, and get the radial components of the magnetic field at different distances from the axis in the $z$ direction are obtained, as shown in Fig. 3.

**Figure 4:**
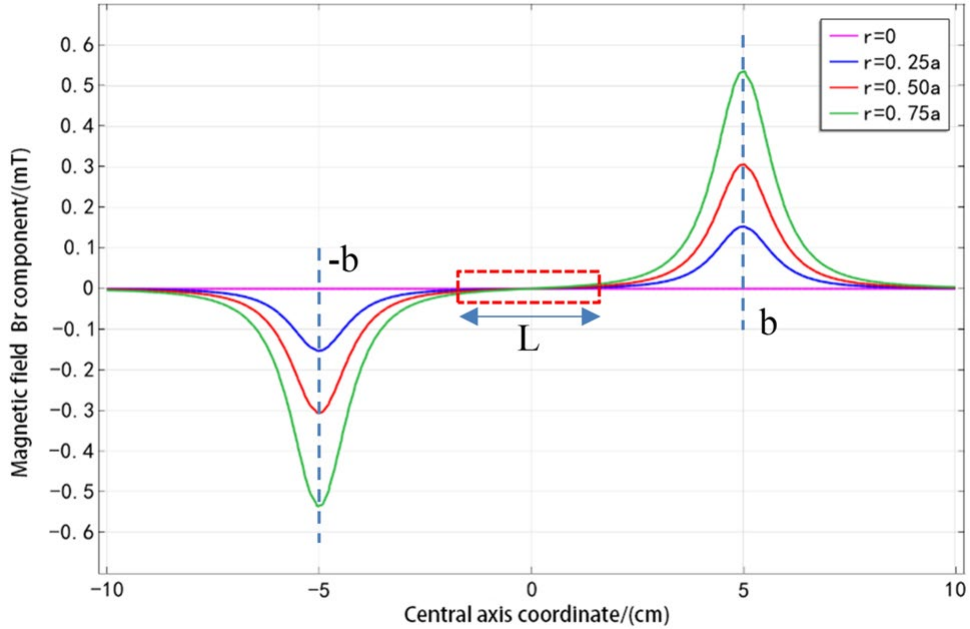
The distribution of $B_{r}$ for different $r$ conditions.

As can be seen from Fig. 4, when $r = {0}$, the component $B_{r}$ on the central axis is zero. When $r$ is not equal to zero, there is a component $B_{r}$, and within the radius of the solenoid, the radial component $B_{r}$ increases with the increase of the distance $r$. The distribution characteristics in the $z$ direction are as follows. With the increase of the $z$ coordinates, the $B_{r}$ value first increases from zero and then decreases to zero, and there is a maximum at both ends of the solenoid $z = \pm b$, but the value of $B_{r}$ is much smaller than that of $B_{z}$, especially for the range of the inner middle section $\left( { - L/2 < z < L/2} \right)$ of the solenoid, and the component $B_{r}$ is almost zero.

It can be concluded that the radial component of the magnetic field is often neglected and only the axial component is considered in the analysis of the magnetic field in the solenoid. This conclusion can bring great convenience to the actual analysis and calculation, and the error caused by the simplification can be ignored.

### Magnetic field of multi-layer solenoid

In practical applications, in order to increase the magnetic field and obtain a larger Faraday rotation angle, multi-layer solenoids are often used without increasing the output power of the modulation signal and the volume of the solenoid. According to the Biot–Savart law and the magnetic field superposition principle, the magnetic field of a multi-layer solenoid can be regarded as the superposition of the magnetic fields of multiple single-layer solenoids, but the thickness of the multi-layer coil cannot be ignored here. so, the equivalent radius should be introduced to replace the previous radius.

Here, only the axial component of the magnetic field on the central axis is discussed. From Eq. (14), it can be obtained that the magnetic field in the multi-layer solenoid is

16 $$B_{z} \left( {0,z} \right) = \frac{{\mu_{0} nI}}{2}\left( {\frac{z + b}{{\sqrt {a_{{\text{d}}}^{2} + \left( {z + b} \right)^{2} } }} - \frac{z - b}{{\sqrt {a_{{\text{d}}}^{2} + \left( {z - b} \right)^{2} } }}} \right) \cdot N_{c} .$$

where $N_{c}$ is the number of layers of the coil, and $a_{{\text{d}}} = (a_{2} - a_{1} )/\ln (a_{2} /a_{1} )$ is the equivalent radius, where $a_{{1}}$ is the inner diameter of the coil, and $a_{{2}}$ is the outer diameter of the coil. In fact, the magnetic field of a multi-layer solenoid is similar to that of a single-layer solenoid. For the analysis of the magnetic field near the axis, there is only a multiple relationship.

In “System principle”, the magneto-optical modulation system introduced in the principle of the system uses a multi-layer solenoid, and its basic parameters are: solenoid length ${2}b = {\text{10 cm}}$, coil inner diameter $a_{{1}} = {3}{\text{.5 cm}}$, outer diameter $a_{{2}} = {4}{\text{.0 cm}}$, number of layers $N_{c} = {5}$, and coil density $n = {1000}$. The signal generator is connected to the power amplifier to output direct current (DC) excitation; the excitation voltage is ${\text{5 V}}$, and the magnetic field of each position on the solenoid axis is measured with a Gauss meter, in order to ensure the accuracy of measurement and positioning, the wooden ruler is used for accurate measurement, as shown in Table 1.

**Table 1:** Measurement of magnetic field at each position on the axis.

Position z/(cm)	− 13	− 9	− 5	− 4	− 3	− 2	− 1	0	1	2	3	4	5	9	13
Measure1	0.26	0.76	2.88	3.66	4.32	4.76	4.89	5.08	5.01	4.74	4.32	3.6	2.98	0.74	0.23
Measure2	0.22	0.82	2.92	3.73	4.3	4.72	4.92	5.02	4.94	4.81	4.28	3.65	2.93	0.72	0.28
Measure3	0.29	0.79	2.90	3.68	4.00	4.70	4.96	5.04	4.99	4.79	4.36	3.68	2.96	0.69	0.26
Average Bz/(mT)	0.26	0.79	2.90	3.69	4.21	4.73	4.92	5.05	4.98	4.78	4.32	3.64	2.96	0.72	0.26

The position in Table 1 represents the distance between the measuring point and the center of solenoid, and positive and negative represents that the measuring position is on both sides and symmetrical about the center. Measurement 1, 2 and 3 represent three rounds of measurement. The order of measurement points is from zero to the right (positive direction) until the coordinate is the position $13\,{ cm}$, and then from the negative direction of zero. The specific location and value of measurement as shown in Table 1. Finally, the average of the three measurements is taken as the final result of the measurement.

**Figure 5:**
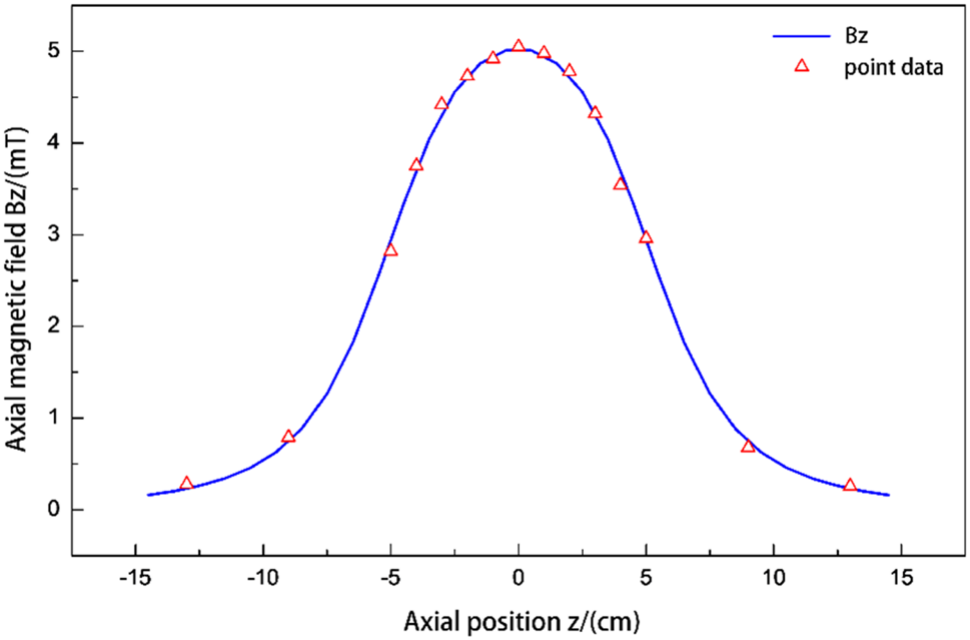
The magnetic field on the central axis.

According to Eq. (16) and the data in Table 1, the calculation is as shown in Fig. 5.

The solid blue line in Fig. 5 represents the curve obtained from the theoretical model, and the small red triangle is the measured value of several points in Table 1. By observing the data and the numerical curves in the table, it can be seen that the magnetic field intensity at the center is the largest. As the distance from the center increases, the magnetic field becomes smaller. When the position is $z = \pm {\text{5 cm}}$ at both ends of the solenoid, the magnetic field amplitude is almost half of the central value, and when the position is $z = \pm {\text{15 cm}}$ at the end of the solenoid, the magnetic field is almost zero. The measured data points are in good agreement with the theoretical curve, which shows that it is feasible to use this method to calculate the magnetic field of multi-layer solenoid.

Therefore, without considering other factors, the single-layer solenoid can usually be studied first, and then, the magnetic field of the multi-layer solenoid can be calculated with the superposition principle and the equivalent radius method, which can simplify the solution of the magnetic field and is suitable for most cases.

## Analysis of solenoid alternating magnetic field

In the process of magneto-optical modulation, the current signal supplied to the solenoid coil is an AC signal, usually a sinusoidal signal. The magnetic field generated by the coil excited by the AC signal is called a time-varying electromagnetic field or an alternating electromagnetic field. The research on the alternating electromagnetic field of a solenoid is the basis for solving the magnetic field in a solenoid.

### Theoretical model of time-varying electromagnetic field

It is assumed that an alternating current $I_{s} = I_{m} e^{{ - {\text{i}}\omega t}}$ is applied to the solenoid coil, that is, a sinusoidal signal with amplitude $I_{m}$ and frequency $\omega$. The line current of the close-wound solenoid is equivalent to the cylindrical current. Based on the symmetry analysis, the components $B_{\varphi }$, $E_{z}$, and $E_{r}$ do not exist in the solenoid. At this point, the electromagnetic field inside the solenoid can be expressed as

17 $$\left\{ {\begin{array}{*{20}c} {{\mathbf{B}}\left( {r,z,t} \right) = B_{r} \left( {r,z,t} \right){\mathbf{r}} + B_{z} \left( {r,z,t} \right){\mathbf{z}}} \\ {{\mathbf{E}}\left( {r,z,t} \right) = E_{\varphi } \left( {r,z,t} \right){\mathbf{\varphi }}} \\ \end{array} } \right.,$$

where ${\varvec{B}}$ and ${\varvec{E}}$ represent the magnetic field and electric field of the solenoid, respectively, $B_{r}$ and $B_{z}$ are the radial and axial components of the magnetic field, respectively, $E_{\varphi }$ is the circumferential component of the electric field, $z$ is the coordinate of the central axis, $r$ is the distance to the central axis, and $t$ is the time variable.

The Maxwell’s equations describe the relationship between the spatially time-varying electromagnetic fields, satisfying the following differential form:

18 $$\left\{ {\begin{array}{*{20}c} {\nabla \times {\mathbf{E}} = - \frac{{\partial {\mathbf{B}}}}{\partial t}} \\ {\nabla \times {\mathbf{H}} = {\mathbf{J}} + \frac{{\partial {\mathbf{D}}}}{\partial t}} \\ {\nabla \cdot {\mathbf{D}} = \rho } \\ {\nabla \cdot {\mathbf{B}} = 0} \\ \end{array} } \right.,$$

where ${\varvec{J}}$ is the current density, $\rho$ is the charge density, ${\mathbf{D}} = \varepsilon_{0} {\mathbf{E}}$ is the potential shift vector, $\varepsilon_{0}$ is the vacuum dielectric constant, ${\mathbf{B}} = \mu_{0} {\mathbf{H}}$, and $\mu_{0}$ is the vacuum permeability.

For the region ${\varvec{J}} = {0}$ and $\rho = 0$ inside the solenoid $\left( {r < a} \right)$, the electromagnetic field expression is replaced with Maxwell’s equations, and the relationship between the components of the magnetic field and the electric field is as follows:

19 $$\frac{{\partial E_{\varphi } }}{\partial z} = - {\text{i}}\omega B_{r} .$$

20 $$\frac{1}{r}\frac{{\partial \left( {rE_{\varphi } } \right)}}{\partial r} = {\text{i}}\omega B_{z} .$$

21 $$\frac{{\partial B_{r} }}{\partial z} - \frac{{\partial B_{z} }}{\partial r} = - {\text{i}}\omega \mu_{0} \varepsilon_{0} E_{\varphi } .$$

Equations (19–21) are the electromagnetic field relationships in vacuum. Theoretically, they can be used to obtain the full space solution of magnetic field or electric field.

For the practice of magneto-optical modulation, the placement of the magneto-optical glass indicates that the magnetic field inside the solenoid is only effective in the magneto-optical medium region, so, the following is simplified as the solution of the electromagnetic field in the paraxial region. According to the conclusion drawn in “Distribution characteristics of magnetic field in the paraxial region”, the magnetic field component $B_{r}$ in the near axial region can be ignored in the analysis of $B_{z}$. By setting $B_{r} = 0$, and by combining Eqs. (20) and (21), the following expressions can be obtained

22 $$r^{2} \frac{{\partial^{2} B_{z} }}{{\partial r^{2} }} + r\frac{{\partial B_{z} }}{\partial r} + k_{0}^{2} r^{2} B_{z} = 0.$$

23 $$r^{2} \frac{{\partial^{2} E_{\varphi } }}{{\partial r^{2} }} + r\frac{{\partial E_{\varphi } }}{\partial r} + (k_{0}^{2} r^{2} - 1)E_{\varphi } = 0.$$

Equations (22) and (23) are the basic models for solving the electromagnetic field in the paraxial region of an AC solenoid. In terms of expression, they are the zero-order and first-order modified Bessel functions. In the equations, $r$ represents the distance from the central axis, and $k_{0} = \omega \sqrt {\mu_{0} \varepsilon_{0} } = \omega /c$ is the wave vector in vacuum.

### Electromagnetic field of a hollow solenoid

A solenoid without a magneto-optic medium is called a hollow solenoid, and the magnetic and electric fields of a hollow solenoid are solved with the time-varying electromagnetic field model.

The general solution of the axial magnetic field $B_{z}$ inside the solenoid is obtained by solving the Bessel function for Eq. (22)

24 $$B_{z} \left( r \right) = A{\text{J}}_{0} \left( {k_{0} r} \right) + C{\text{N}}_{0} \left( {k_{0} r} \right),$$

where $A$ and $C$ are the integral constants to be solved, ${\text{J}}_{0} \left( {k_{0} r} \right)$ is the first kind of zero-order Bessel function, and ${\text{N}}_{0} \left( {k_{0} r} \right)$ is the second kind of zero-order Bessel function.

The values of the constants $A$ and $C$ are determined, and the relationship with the size of the magnetic field is examined when $\omega \to 0$. Because the relationship $B_{z} \left( {\omega \to 0} \right) = A{\text{J}}_{0} \left( {k_{0} r \to 0} \right) + C{\text{N}}_{0} \left( {k_{0} r \to 0} \right) = B_{0}$, ${\text{J}}_{0} \left( {k_{0} r \to 0} \right) = 1$ and ${\text{N}}_{0} \left( {k_{0} r \to 0} \right) \to - \infty$, so, $A = B_{0}$, $C = 0.$ Therefore,

25 $$B_{z} \left( {r,t} \right) = B_{0} {\text{J}}_{0} \left( {k_{0} r} \right) \cdot e^{{ - {\text{i}}\omega t}} ,$$

where $B_{0}$ is the magnetic field inside the solenoid when excitation is provided by a DC electric signal.

Similarly, the solution of the circumferential electric field inside the solenoid is obtained with Eq. (23)

26 $$E_{\varphi } \left( r \right) = A^{\prime}{\text{J}}_{1} \left( {k_{0} r} \right) + C^{\prime}{\text{N}}_{1} \left( {k_{0} r} \right),$$

where $A^{\prime}$ and $C^{\prime}$ are integral constants, ${\text{J}}_{1} \left( {k_{0} r} \right)$ is a first-order Bessel function of the first kind, and ${\text{N}}_{1} \left( {k_{0} r} \right)$ is a second-order Bessel function of the second kind. Taking the same $\omega \to 0$, here, ${\text{J}}_{1} \left( {k_{0} r \to 0} \right) = 0$, ${\text{N}}_{1} \left( {k_{0} r \to 0} \right) \to - \infty$, so, $C^{\prime} = 0$. The value of $A^{\prime}$ can be determined with the following relationship:

27 $${\mathbf{E}} = \frac{{\text{i}}}{\omega \mu \varepsilon }\nabla \times {\mathbf{B}} = - \frac{{\text{i}}}{\omega \mu \varepsilon }\frac{{{\text{d}}B}}{{{\text{d}}r}}{\mathbf{e}}_{\varphi } .$$

$A^{\prime} = {\text{i}}cA$ is obtained with Eqs. (25–27), and $c$ is the speed of light. Hence, the electric field is expressed as

28 $$E_{\varphi } \left( {r,t} \right) = {\text{i}}cB_{0} J_{1} \left( {k_{0} r} \right) \cdot e^{{ - {\text{i}}\omega t}} .$$

**Figure 6:**
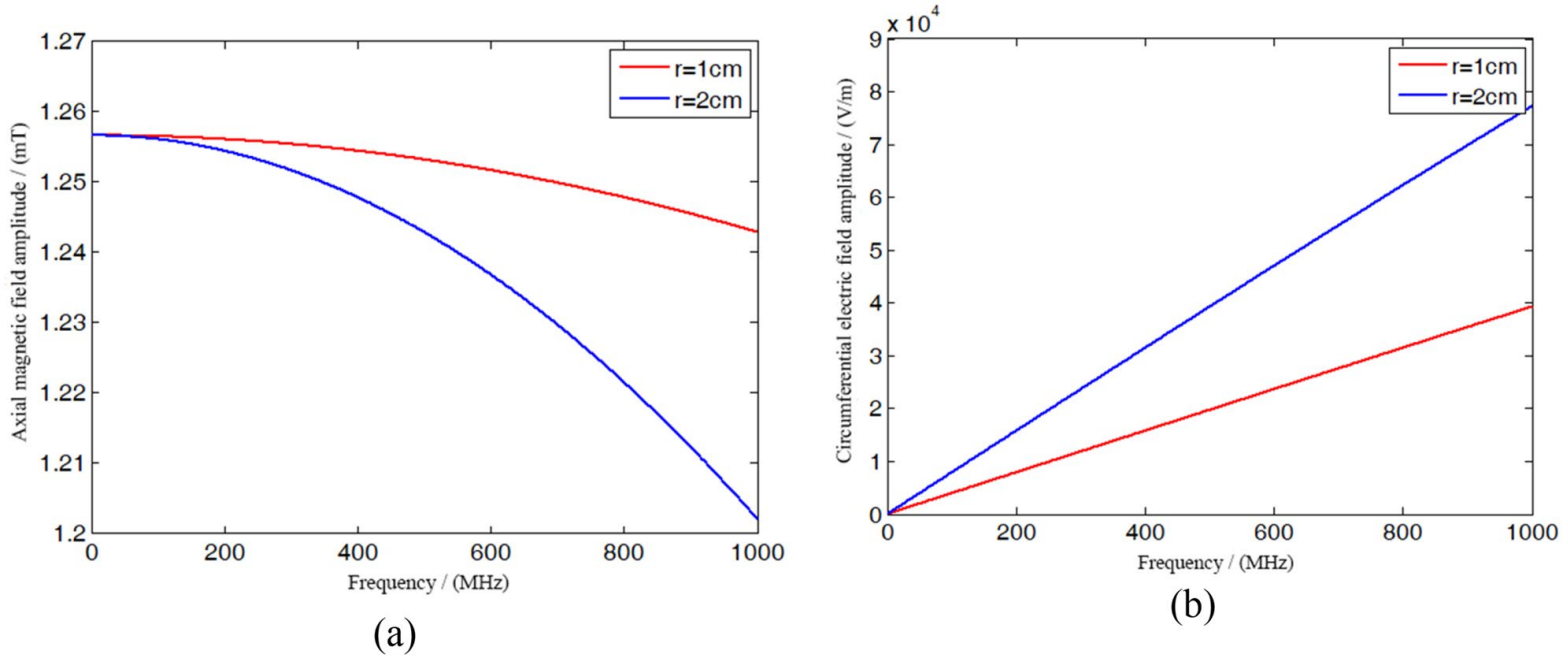
The amplitude of the alternating electromagnetic field in the solenoid varying with the frequency. (**a**) Magnetic field; (**b**) electric field.

Equations (25) and (28) are the expressions of the AC electromagnetic field inside the hollow solenoid, and these expressions have the same sinusoidal variation form as the exciting current. It can be seen that the alternating electromagnetic field is closely related to the frequency. The change of frequency will affect the change of the numerical value, and this change will in turn be affected by the position. The relationship between the electromagnetic field amplitude and frequency is shown in Fig. 6.

Figure 6 shows the frequency dependence of the amplitude of the alternating electromagnetic field in the solenoid. It can be seen from Fig. 6a that when the AC frequency is less than $100\,{ MHz}$, the amplitude of the AC magnetic field hardly changes, because the additional magnetic field excited by the low frequency AC electric field is very weak. When the frequency is high, the amplitude attenuation is obvious, and the magnetic field excited by the high-frequency AC electric field cannot be ignored. Compared with the two curves, the amplitude of the axial magnetic field represented by the curves decays with the increase of the frequency, but the magnetic field far away from the axis $\left( {r = 2\,{ cm}} \right)$ decays faster, indicating that the magnetic field closer to the center is less susceptible to the change of frequency. It can be seen from Fig. 6b that the two curves show an increasing trend from zero, indicating that the alternating magnetic field stimulates the induced electric field, and the electric field amplitude increases with the increase of frequency. Similar to the change of the magnetic field, the electric field far from the axis changes faster, which indicates that the closer the position is to the center, the better the stability of the electromagnetic field.

## Analysis of magnetic field influence on azimuth transmission system

It can be seen from the previous analysis that the Verdet constant $V$ and the magnetic induction $B$ of the material are affected by many factors, except for $L$ in the fixed and directly measurable quantity of the modulation $m_{f} = 2VBL$. As a result, the modulation $m_{f} = 2VBL$ changes, and then, the azimuth calculation model including the modulation $m_{f}$ changes, which affects the accuracy of the non-line-of-sight azimuth transmission system.

First, we focus on the Verdet constant of magneto-optical glass. In order to better interpret the essence of the Tb_3_Ga_5_O_12_ (TGG) Verdet constant of the magneto-optic glass, the wave-particle duality theory of light is introduced. Light has both wave characteristics and particle characteristics, and the two characteristics are equally significant and exist simultaneously in the propagation process^14^. Based on this idea, considering the fact that the classical theory reflects the wave properties of light, and the electric dipole transition explained by the quantum theory reflects the particle properties of light, the conjecture hypothesis is put forward: the two parts of the effects explained by the classical theory and the quantum theory exist at the same time, acting on the Faraday rotation together. The Faraday effect can be explained in two parts. One part is caused by the volatility of light, which makes the Faraday rotation angle positive, corresponding to the positive component of the Verdet constant. The other part is caused by the particle effect of light, which makes the rotation angle negative. Correspondingly, the Verdet constant component is negative. Therefore, the deviation of the calculation result in the classical theory is not affected by the efficiency equal multiplicative factor but rather derived from the additive component produced by the quantum effect. Based on the above conjecture, the theory of the contribution of wave transition is proposed, that is, the Faraday magneto-optical effect is the result of the interaction of light waves and matter (wave) and the excited transition of the electric dipole (transition). The fluctuation and transition contribute to the Faraday angle at the same time, and the magnitude of the contribution is related to the magnetic properties of the material, the structural properties of the material, the major contributing ions, the wavelength of incident light, and the temperature of the material.

Based on the dielectric dispersion equation, the fluctuation contribution of the TGG Verdet constant is calculated, and its refractive index satisfies the Sellmeier formula^15^: $n^{2} \left( \lambda \right) = {A}_{1} + \frac{{{B}_{1} }}{{\lambda^{2} }} + \frac{{{C}_{1} }}{{\lambda^{4} }}$, where ${A}_{1} = 3.734$, ${B}_{1} = 48210\,{ nm}^{2}$, and ${C}_{1} = 1.178 \times 10^{8} \,{ nm}^{4}$.

According to the theory of volatility transition contribution, the volatility contribution term of the paramagnetic material magneto-optical glass TGG Verdet constant is:

29 $$V_{l} \left( {{\text{TGG}}} \right) = 293.3\frac{{{\text{B}}_{1} \lambda^{2} + 2{\text{C}}_{1} }}{{\lambda^{2} \sqrt {{\text{A}}_{1} \lambda^{4} + {\text{B}}_{1} \lambda^{2} + {\text{C}}_{1} } }} \cdot \left( {\frac{T}{T + 7}} \right),$$

The transitional contribution term of the paramagnetic material magneto-optical glass TGG Verdet constant is:

30 $$V_{h} (\text{{TGG}}) = \frac{{1.504 \times 10^{10} }}{{\left( {\lambda^{2} - 63605} \right)\left( {T + 7} \right)}},$$

The Verdet constant of the paramagnetic material magneto-optical glass TGG is:

31 $$V\left( {{\text{TGG}}} \right) = 293.3\frac{{{\text{B}}_{1} \lambda^{2} + 2{\text{C}}_{1} }}{{\lambda^{2} \sqrt {{\text{A}}_{1} \lambda^{4} + {\text{B}}_{1} \lambda^{2} + {\text{C}}_{1} } }} \cdot \left( {\frac{T}{T + 7}} \right) + \frac{{1.504 \times 10^{10} }}{{\left( {\lambda^{2} - 63605} \right)\left( {T + 7} \right)}}.$$

**Figure 7:**
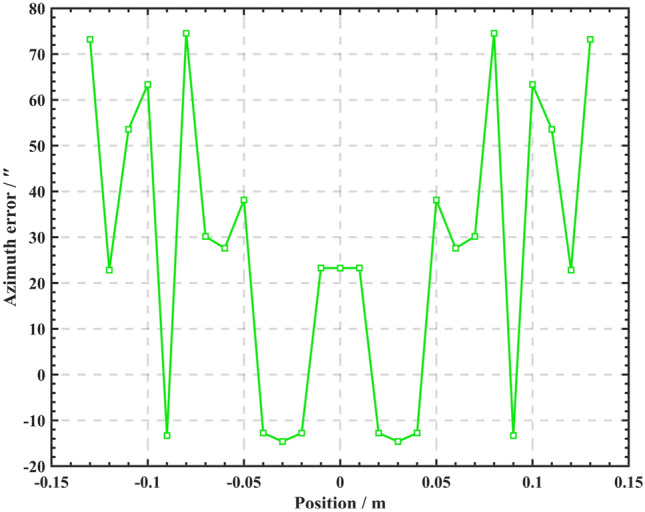
Azimuth transmission error diagram.

The influence of the Verdet constant can be further analyzed according to Eq. (31). This article mainly describes the study of a solenoid magnetic field. According to the research results, combined with the solenoid used in the non-line-of-sight azimuth transmission system, the change results of the magnetic field of the multi-layer solenoid described in “Magnetic field of multi-layer solenoid” are selected, and the results of the influence of the magnetic field are substituted into the modulation $m_{f}$. According to the azimuth misalignment angle model derived from Eq. (5), with the help of the numerical simulation software MATLAB, the azimuth transfer error curve for the change of the internal magnetic field of the multi-layer solenoid is obtained, as shown in Fig. 7.

In Fig. 7, the abscissa is the corresponding position in Fig. 5 of “Magnetic field of multi-layer solenoid” in this paper, that is, the symmetrical position along the central axis $- 0.13\,{ m} \sim + 0.13\,{ m}$ of the solenoid, The ordinate is the azimuth error caused by the magnetic field at different positions of the central axis. It can be seen from Fig. 7 that when the magneto-optical modulator works at room temperature, the azimuth transfer error is symmetrically distributed with the center position of the solenoid containing the magneto-optical medium, the azimuth transfer error of solenoid center position is about 22″. As the position moves slightly left or right along the center, the azimuth transfer error first decreases and then increases, in the position $- 0.04\,{ m} \sim + 0.04\,{ m}$ of the central axis of the solenoid, the azimuth error is within the acceptable range (20″), when the position exceeds 0.04 m and is in $- 0.08\,{ m} \sim - 0.13\,{ m}$ and $+ 0.08\,{ m} \sim + 0.13\,{ m}$, the azimuth error changes dramatically and produces the maximum azimuth error. According to the principle derivation and simulation results, the solenoid containing magneto-optical medium seriously restricts the azimuth transmission accuracy of the system. It can be seen from Fig. 5 that the position far away from the center point of the solenoid has a significant attenuation of the magnetic field and a significant increase of the azimuth transfer error.

## Conclusion

This paper mainly describes the study of the magnetic field produced by an electrified solenoid and discusses the static and alternating solenoid magnetic fields. First, a solenoid magnetic field excited by direct current is analyzed, the theoretical model of the magnetic field is deduced, and the amplitude and distribution of the magnetic field are analyzed. Then, the distribution characteristics of the magnetic field in the near axis region of solenoid are mainly discussed. Generally, the magnetic field in the near axis region is uniform and along the axial direction. The magnetic field of the multi-layer solenoid can be obtained with the superposition principle. Finally, the solenoid excited by the alternating current is analyzed. Based on Maxwell’s equation, the alternating magnetic field model is established. It is pointed out that the amplitude of the magnetic field will have an attenuation effect for high-frequency excitation. The magnetic field characteristics of a hollow solenoid are discussed. For the magnetic field in the solenoid with an alternating current, the following conclusions are drawn: (1) for the magnetic field inside the solenoid not close to the end face, only considering the axial component can bring great convenience for the actual analysis and calculation, and the error can be ignored. (2) The magnetic field in the solenoid is the superposition of a series of additional magnetic fields induced by the induced electric field. The total magnetic field after superposition has the same change rule as the excitation current. (3) Frequency is the main factor that affects the magnetic field. The amplitude of the magnetic field will decrease with the increase of the frequency. For the condition of low frequency (frequency less than $100\,{MHz}$), there is almost no attenuation of the magnetic field amplitude, and it is considered that the magnetic field is uniformly distributed in the radial direction. (4) According to the analysis of system influence, both the Verdet constant of magneto-optical material and the magnetic field of solenoid strictly limit the accuracy of magneto-optical modulator, which further affects the function of magneto-optical modulator in non-line-of-sight azimuth transmission system, resulting in the fluctuation of system accuracy. Therefore, only by fully verifying and optimizing the magneto-optical modulator can the accuracy of non-line-of-sight azimuth transmission system be effectively improved. According to the theoretical derivation and experimental verification, the research results can be used as an effective reference to improve the accuracy of non-line-of-sight azimuth transmission system.
